# Zinc status in attention-deficit/hyperactivity disorder: a systematic review and meta-analysis of observational studies

**DOI:** 10.1038/s41598-021-94124-5

**Published:** 2021-07-16

**Authors:** Seyed Mojtaba Ghoreishy, Sara Ebrahimi Mousavi, Farzaneh Asoudeh, Hamed Mohammadi

**Affiliations:** Department of Clinical Nutrition, School of Nutritional Sciences and Dietetics, Tehran University of Medical Sciences, Tehran, Islamic Republic of Iran

**Keywords:** Neuroscience, Neurological disorders, Malnutrition

## Abstract

Previous studies regarding the zinc status in attention-deficit/hyperactivity disorder (ADHD) yielded inconsistent results. Thus, the present meta-analysis was aimed to estimate the association between hair and serum/plasma zinc levels and ADHD. Online databases of Medline, EMBASE, and Scopus were searched up to October 2020 with no limitation in time and language. Weighted mean differences (WMDs) of hair and serum/plasma zinc levels were calculated using a random-effects model. Overall, 22 articles with 1280 subjects with ADHD and 1200 controls were included. The pooled effect size indicated that serum/plasma zinc levels in subjects with ADHD were not statistically different than their controls (WMD = − 1.26 µmol/L; 95% CI − 3.72, 1.20). Interestingly, the exclusion of one study from the analysis showed that people with ADHD significantly have lower circulating levels of zinc compared to their controls (WMD: − 2.49 µmol/L; 95% CI − 4.29, − 0.69). Also, the pooled effect size indicated that hair zinc levels in cases with ADHD were not statistically different than their controls (WMD = − 24.19 μg/g; 95% CI − 61.80, 13.42). Present meta-analysis raises the possibility that subjects with ADHD are prone to have declined levels of zinc levels. Based on current findings, screening the zinc levels in subjects with ADHD could be reasonable. Further well-designed studies are needed to clarify the role of zinc in the etiology of ADHD.

## Introduction

Attention-deficit/hyperactivity disorder (ADHD) is a common neurodevelopmental disorder that affects approximately one in every ten children^1^. ADHD imposes a significant health and financial burden on patients, society, and the health care system^2,3^. Impulsivity decreased concentration and social interaction difficulties are the main manifestations of ADHD^4^.

The etiology of ADHD is multifactorial, including a contribution of genetic and environmental factors, perinatal risks, and pollutant exposure^5,6^. Several reports suggested the importance of vitamins and minerals in ADHD development and symptoms^6,7^. Both deficiency and excesses of minerals have been shown in relation to ADHD^8^. Among the trace elements zinc plays a crucial role in the etiology of ADHD ^9^. In ADHD, the dopaminergic and adrenergic systems are disrupted^10^. Zinc is involved in melatonin production which modulate the function of dopamine and facilitate dopamine signaling^11^.

Results of previous reports regarding the relationship between zinc levels and ADHD are inconsistent. Tippairote et al. in a case–control study, showed that higher hair zinc level was associated with greater ADHD symptoms and inattention^12^. On the other hand, Skalny et al. showed that lower levels of zinc and magnesium may significantly contribute to the severity of ADHD symptoms^13^. Also, several interventional studies suggested that zinc supplementation is effective in the improvement of ADHD symptoms^14,15^.

Previously, Luo et al. in a meta-analysis on 11 observational studies quantified the association between zinc levels and ADHD. The results showed no significant association between blood and hair zinc levels with ADHD^16^. A small sample size was one of the major limitations of this study and the large number of cases may increase the statistical power to clarify the relationship between zinc status and ADHD. Since that time, 11 additional studies have been published. Therefore, we conducted an updated meta-analysis, including more recent data, to provide quantitative estimates of the association between zinc status and ADHD.

## Methods

The present study conducted based on the Preferred Reporting Items for Systematic Reviews and meta-analyses (PRISMA) statement^17^.

### Search strategy

Electronic searches of PubMed, EMBASE, and Scopus were conducted up to October 2020 with no limitation in time and language. To search for titles, abstracts, and keywords of articles, a search was performed using **“**Zinc” OR “Trace Elements” OR “Trace Element*” AND “Attention Deficit Disorder with Hyperactivity” OR “ADHD”.

### Study selection

After removing the duplicate studies, the title and abstract of the remaining studies were screened by two independent researchers (SMG and SEM). Finally, the full text of the relevant articles was reviewed and any discrepancy was resolved with the consensus of the researchers. All observational studies that examined peripheral levels of zinc (including blood and hair) between ADHD and control were included. We excluded trial and cohort studies, conference abstracts, letters, notes, editorials, reviews, or meta-analysis.

### Data extraction

SMG and SEM extracted the required information from the included studies. Any disagreements were resolved by discussion or if necessary, by the third investigator (HM). Extracted information included: name of first author, publication year, country, sample size, mean age, body mass index, method of zinc assessment, criteria of ADHD diagnosis, the mean and corresponding standard deviation of zinc, and study design.

### Quality assessment

Newcastle–Ottawa Scale (NOS) was used to evaluate the quality of the included studies. Articles with a total score of 0–4, 5–7, and 8–10 were considered as low, moderate and high quality, respectively.

### Statistical analysis

To merge data, we used the random-effects model. In order to the calculation of effect size, the concentrations of zinc were converted to µmol/L. Heterogeneity of studies is determined using I^2^ and Chi-square test, high heterogeneity is determined by I^2^ above 50% and *P* value < 0.1. In order to determine the origin of heterogeneity, we used subgroup analysis and sensitivity analysis. Subgroup analysis was performed according to the type of samples (serum, plasma, blood), year of publication (≤ 2010, > 2010), method of zinc assessment (atomic absorption spectrophotometer, others), study design (case–control, cross-sectional), and sample size (< 100, > 100). Begg’s and Egger’s tests were used to examine the publication bias. STATA software (version 14)^18^ was used for statistical analysis. Statistically significance is confirmed with *P* value less than 0.05.

## Results

### Study selection

**Figure 1:**
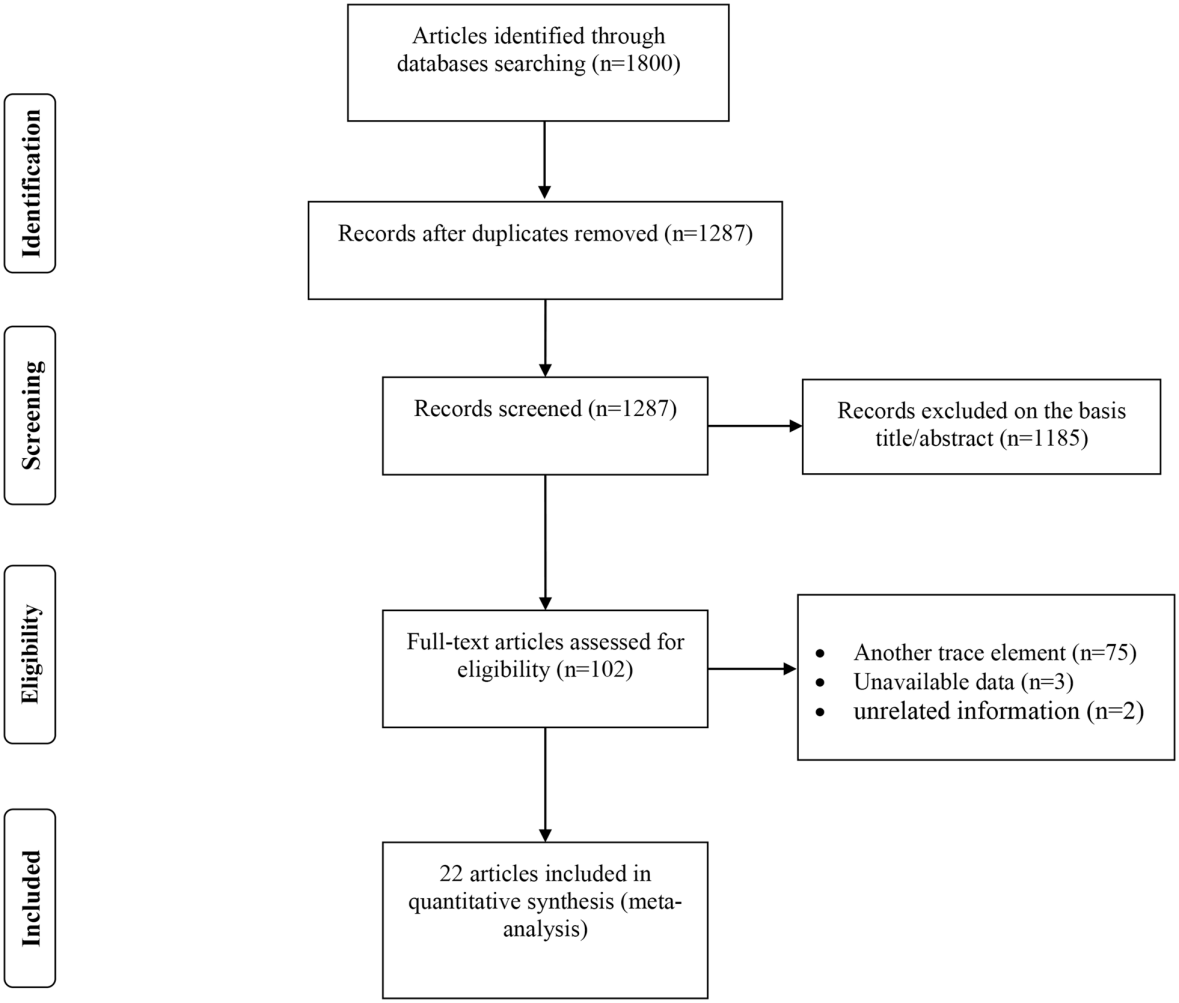
PRISMA flowchart describing the study’s systematic literature search and study selection.

The study selection process is illustrated in Fig. 1. Among the 1800 studies found based on an electronic and manual search for all trace elements, 513 studies were duplicate. Screening the title and abstracts excluded 1185 documents. Among the remaining 102 articles 75 studies were related to other trace elements. Three studies were excluded due to unavailability of information regarding zinc levels^19–21^ and two studies were excluded due to lack of reporting information in case and control groups separately and reporting the level of zinc in hemoglobin^22,23^. Finally, 22 articles were included in this meta-analysis, 14 studies assessed circulating levels of zinc^13,24–36^ and 8 studies reported hair zinc levels^12,37–43^.

### Characteristics of included studies

Characteristics of the included studies were summarized in Table 1. Included studies were conducted between 1990 and 2020, examining 1280 people with ADHD and 1200 controls. Except for one study^24^ in which both case and control groups had diabetes, other studies used healthy individuals and people with ADHD as control and case groups, respectively. Of the available studies, four were conducted in Egypt^24,28,29,33^, three in Russia^13,37,38^, two in Turkey^32,36^, two in the United States^34,41^, and others in Syria^25^, China^26^, Iran^27^, Slovakia^30^, Israel^31^, Saudi Arabia^35^, New Zealand^39^, Indonesia^40^, Thailand^12^, Korea^42^, and Georgia^43^. Twenty studies had case–control design and only two studies^13,28^ were conducted by cross-sectional design. According to the Newcastle–Ottawa scale, nine studies were assigned to moderate quality^12,25,27,29,30,32,36,40,41^, and the rest of them had high quality (Table 2).

**Table 1:** Baseline characteristic of included studies.

First author (year; location)	Study design	Sample	Criteria of ADHD	Population		Sample size	Matching	Mean age (years)	Method of assessment	Nos
				Case	Control	Case\control				
Sakhr (2020; Egypt)	cc	Serum	DSM-IV	T1DM with ADHD	T1DM without ADHD	20\40	Age, sex	Case:10.19 ± 2.34 Control:10.35 ± 3.29	Spectrophotometer	8
Hawari (2020; Syria)	cc	Serum	DSM-V	ADHD	Healthy	29\30	NR	NR	Spectrophotometer by colorimetric assay	7
Yang (2019; China)	cc	Serum	DSM-V	ADHD	Healthy	419\395	BMI z-score	Case: 8.8 ± 2.1 Control: 8.9 ± 1.7	Atomic absorption spectrometry	8
Tinkov (2019; Russia)	cc	Serum	ICD-10	ADHD	Healthy	68\68	Age, gender	Case :6.4 ± 2.1 Control:6.4 ± 2.1	Inductively-coupled plasma mass spectrometry	9
Avval (2019; Iran)	cc	Serum	DSM-IV	ADHD	Healthy	36\15	Age, gender, family history of anemia	Case:7.8 ± 2.12 Control:8.4 ± 3.11	Nr	7
Abdelnaby (2018; Egypt)	cs	Serum	DSM-IV	ADHD	Healthy	25\25	NR	Case:4.00 ± 2.47 Control:5.66 ± 3.9	Routine kinetic and fixed-rate colorimetric methods	6
Elbaz (2017; Egypt)	cc	Serum	DSM-IV-R	ADHD	Healthy	20\20	Age, sex	Case:7.74 ± 1.48 Control:7.40 ± 1.35	Nr	7
Viktorinova (2016; Slovakia)	cc	Plasma	ICD-10	ADHD	Healthy	58\50	NR	Case:9.4 ± 2.1 Control:8.9 ± 2.8	Atomic absorption spectrometry	7
Sandyk (1990; Israel)	cc	Serum	DSM-III-R	ADHD	Healthy	43\28	age	Case:10.1 ± 2.4 Control:11.3 ± 3.2	Atomic absorption spectrophotometry	8
Bekaroglu (1996; Turkey)	cc	Serum	DSM-III-R	ADHD	Healthy	48\45	NR	Case:9.2 ± 2 Control: 9.3 ± 2	Atomic absorption spectrophotometer	7
Mahmoud (2011; Egypt)	cc	Serum	DSM-IV	ADHD	Healthy	58\25	Age, sex, socioeconomic state	Case:8.3 ± 1.8 Control:8.6 ± 3.1	Color metric test without desproteinization	9
Antalis (2006; USA)	cc	Serum	CAARS	ADHD	Healthy	12\12	Gender, BMI, smoking	Case:24.37 ± 2.3 Control:22.37 ± 2.4	Inductively coupled plasma spectrophotometry	9
Khan (2017; KSA)	cc	Plasma	DSM-IV	ADHD	Healthy	41\41	Age, gender	NR	Atomic absorption spectrophotometry	9
Yorbik (2008; Turkey)	cc	Plasma	DSM-IV	ADHD	Healthy	28\24	NR	NR	Atomic absorption spectrophotometry	7
Tinkov (2020; Russia)	cc	Hair	ICD-10	ADHD	Healthy	90\90	Age, gender	Case:5.47 ± 1.57 Control:5.47 ± 1.57	Inductively coupled plasma mass-spectrometry after microwave digestion	9
Skalny (2020; Russia)	cs	Hair	ICD-10	ADHD	Healthy	52\52	Height-weight	Case:5.15 ± 0.97 Control:5.13 ± 1.05	Inductively-coupled plasma mass spectrometry	9
Perham (2020; New Zealand)	cc	Hair	DSM-IV	ADHD	Healthy	55\52	Geographical locations, Socioeconomic backgrounds	Case:9.78 ± 1.56 Control:10.08 ± 1.70	Mass spectrometry and temperature-controlled microwave digestion techniques	9
Setiawati (2019; Indonesia)	cc	Hair	CBRS	ADHD	Healthy	23\21	NR	NR	Atomic absorption spectrophotometry	7
Tippairote (2017; Thailand)	cc	Hair	DSM-V	ADHD	Healthy	45\66	NR	Case:5.56 ± 1.34 Control:5.26 ± 1.29	Inductively coupled plasma mass spectrometry	7
Arnold (1990; USA)	cc	Hair	DSM-III	ADHD	Healthy	18\7	NR	NR	NR	7
Shin (2014; korea)	cc	Hair	DSM-IV	ADHD	Healthy	41\42	Age, gender	Case:115.68 ± 35.67 Control:119.71 ± 34.97	Inductive coupled plasma-mass spectrometry	8
Tabatadze (2018; Georgia)	cc	Hair	DSM-V	ADHD	Healthy	51\52	NR	NR	Roentgen fluorescence spectrometer	6

*BMI* body mass index, *CC* case–control, *CS* cross-sectional, *Zn* zinc, *ADHD* attention deficit hyperactivity disorder.

**Table 2:** Quality assessments of included studies.

Study	Case definition adequate	Representativeness of the cases	Selection of Controls	Definition of Controls	Comparability of cases and controls	Ascertainment of exposure	Same method of ascertainment	Non-response rate	Nos
Sakhr (2020; Egypt)	*	*	*	–	**	*	*	*	8
Hawari (2020; Syria)	*	*	*	*	–	*	*	*	7
Yang (2019; China)	*	*	*	*	*	*	*	*	8
Tinkov (2019; Russia)	*	*	*	*	**	*	*	*	9
Avval (2019; Iran)	*	*	–	*	**	–	*	*	7
Abdelnaby (2018; Egypt)	*	*	–	*	–	*	*	*	6
Elbaz (2017; Egypt)	*	*	–	*	**	–	*	*	7
Viktorinova (2016; Slovakia)	*	*	*	*	–	*	*	*	7
Sandyk (1990; Israel)	*	*	*	*	*	*	*	*	8
Bekaroglu (1996; Turkey)	*	*	*	*	–	*	*	*	7
Mahmoud (2011; Egypt)	*	*	*	*	**	*	*	*	9
Antalis (2006; USA)	*	*	*	*	**	*	*	*	9
Khan (2017; KSA)	*	*	*	*	**	*	*	*	9
Yorbik (2008; Turkey)	*	*	*	*	–	*	*	*	7
Tinkov (2020; Russia)	*	*	*	*	**	*	*	*	9
Skalny (2020; Russia)	*	*	*	*	**	*	*	*	9
Perham (2020; NewZealand)	*	*	*	*	**	*	*	*	9
Setiawati (2019; Indonesia)	*	*	*	*	–	*	*	*	7
Tippairote (2017; Thailand)	*	*	*	*	–	*	*	*	7
Arnold (1990; USA)	*	*	*	*	–	*	*	*	7
Shin (2014; South Korea)	*	*	–	*	**	*	*	*	8
Tabatadze (2018; Georgia)	*	*	–	*	–	*	*	*	6

*NOS* New-castle Ottawa Scale.

### Meta-analysis of mean blood zinc levels

**Figure 2:**
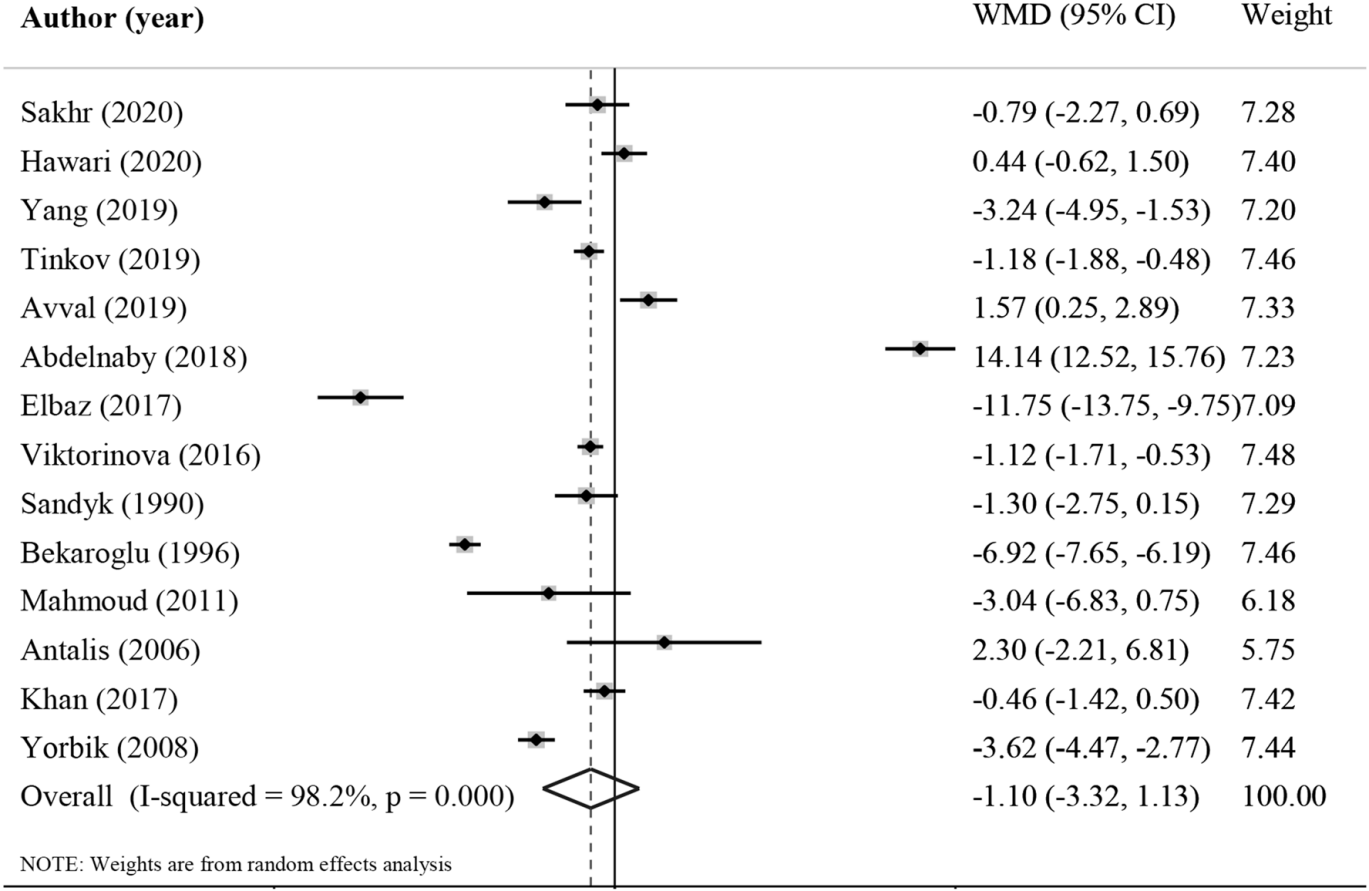
Forest plot for the association between serum zinc level and ADHD expressed as mean difference between case and control groups. The area of each square is proportional to the inverse of the variance of the WMD. Horizontal lines represent 95%Cis. Diamonds represent pooled estimates from random-effects analysis. WMD, weighted mean difference.

Fourteen studies assessed the association between blood zinc level and ADHD^13,24–36^, involving 902 cases and 818 controls. The pooled effect size indicated that serum/plasma zinc levels in subjects with ADHD were not statistically different than their controls (WMD = − 1.26 µmol/L; 95% CI − 3.72, 1.20, *P* = 0.31, Fig. 2). Interestingly, sensitivity analysis showed that exclusion of Abdelnaby's study^28^ from the analysis changed the overall effect size (WMD: − 2.49 µmol/L; 95% CI − 4.29, − 0.69). A significant heterogeneity was detected among studies (I^2^ = 98.2%, P < 0.001). Despite classification of the studies, no possible source of heterogeneity was found and the result remained non-significant in all categories (Table 3). No evidence of publication bias was observed among included studies (*P* = 0.87, Begg’s test and *P* = 0.45, Egger’s test).

**Table 3:** Subgroup analysis to assess the serum and hair zinc levels in subjects with ADHD.

Sub grouped by	No.	WMD (95% CI)	*P* value	P-Heterogeneity	I^2^ (%)	P-between subgroup heterogeneity
**Serum zinc**						
*Sample type*						
Serum	10	− 0.67 (− 4.23, 2.90)	0.714	< 0.001	98.7	
Plasma	3	− 1.73 (− 3.49, 0.02)	0.053	< 0.001	93.2	0.192
Blood	1	− 3.24 (− 4.95, − 1.53)	< 0.001	0	0	
*Publication year*						
≤ 2010	4	− 2.92 (− 5.85, 0.02)	0.052	< 0.001	95.8	< 0.001
> 2010	10	− 0.48 (− 3.05, 2.09)	0.715	< 0.001	98.1	
*Zinc assessment method*						
Atomic absorption spectrophotometer	6	− 2.79 (− 5.04, − 0.53)	0.015	< 0.001	97.3	< 0.001
Other	8	0.23 (− 3.82, 4.28)	0.912	< 0.001	98.4	
*Sample size*						
≤ 100	11	− 0.88 (− 4.14, 2.39)	0.598	< 0.001	98.6	0.004
> 100	3	− 1.49 (− 2.32, − 0.67)	< 0.001	0.066	63.1	
*Study design*						
Case–control	13	− 2.31 (− 3.97, − 0.64)	0.007	< 0.001	96.7	< 0.001
Cross-sectional	1	14.14 (12.52, 15.76)	< 0.001	< 0.001	0	
**Hair zinc**						
*Publication year*						
≤ 2010	1	− 65.10 (− 191.40, 61.20)	0.312	0	0	0.237
> 2010	7	− 4.54 (− 38.76, 29.68)	0.795	< 0.001	98.2	
*Zinc assessment method*						
Atomic absorption spectrophotometer	1	97.33 (33.73, 160.93)	0.003	0	0	0.008
Other	7	− 19.9 (− 54.14, 15.95)	0.286	< 0.001	98.2	
*Sample size*						
≤ 100	5	− 11.74 (− 49.82, 26.33)	0.545	< 0.001	98.8	0.712
> 100	3	− 2.78 (− 119.62, 114.07)	0.963	0.003	82.8	
*Study design*						
Case–control	7	− 11.79 (− 49.59, 26.01)	0.541	< 0.001	98.3	0.544
Cross-sectional	1	17.57 (− 3.18, 38.32)	0.097	0	0	

### Meta-analysis of mean hair zinc levels

**Figure 3:**
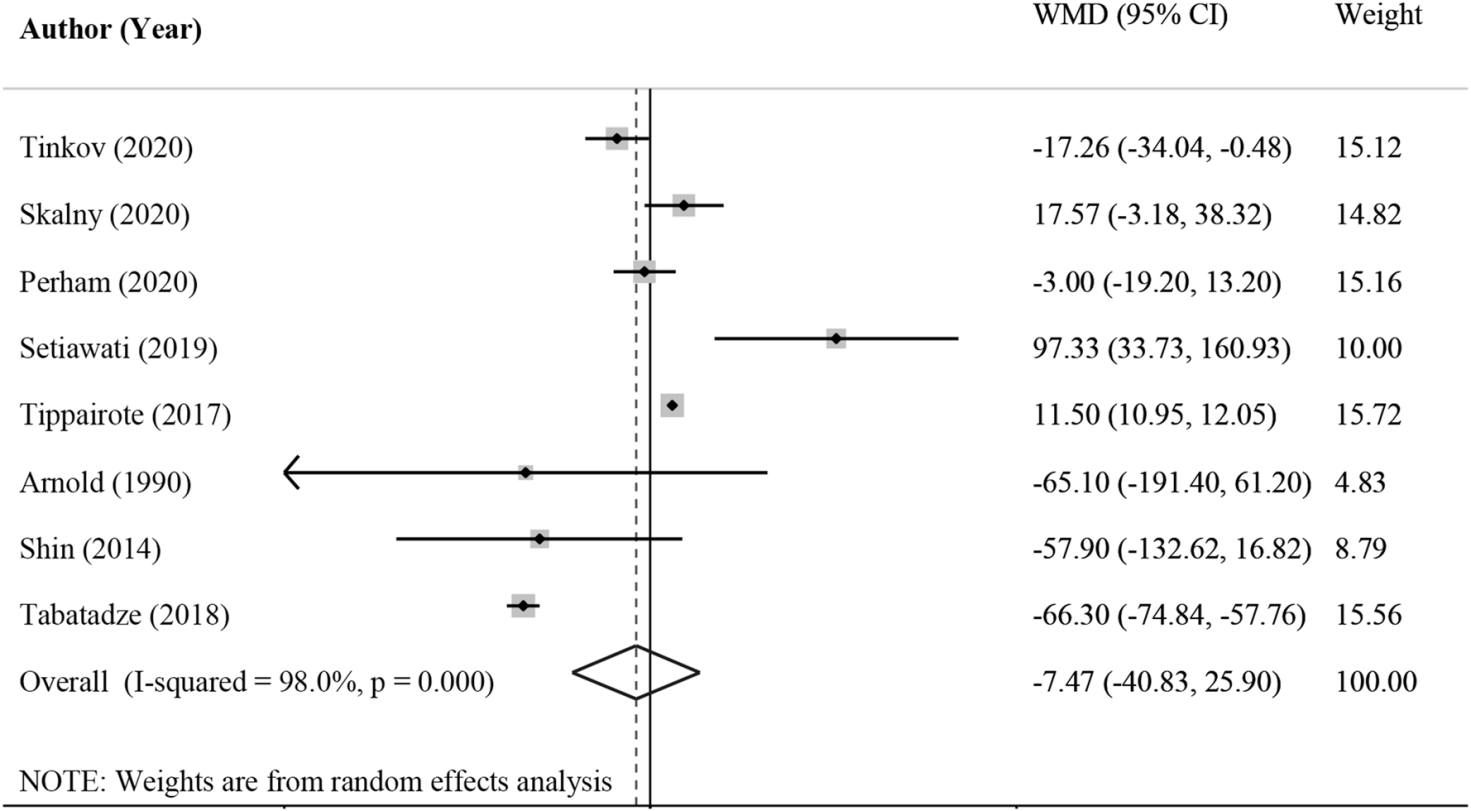
Forest plot for the association between hair zinc level and ADHD expressed as mean difference between case and control groups. The area of each square is proportional to the inverse of the variance of the WMD. Horizontal lines represent 95%Cis. Diamonds represent pooled estimates from random-effects analysis. WMD, weighted mean difference. All statistical analyses were performed using Stata version 14 (StataCorp. 2015. Stata Statistical Software: Release 14. College Station, TX: StataCorp LP, www.stata.com).

Eight studies reported sufficient data regarding hair zinc levels in ADHD and control subjects^12,37–43^, involving 375 cases and 382 controls. The pooled effect size indicated that hair zinc levels in cases with ADHD were not statistically different than their controls (WMD = − 24.19 μg/g; 95% CI − 61.80, 13.42, *P* = 0.20, Fig. 3). However, significant heterogeneity was detected across the studies (I^2^ = 98.1%, *P* < 0.001). Despite the different subgroup analysis, we could not detect the potential source of observed heterogeneity, as shown in Table 2. There was no evidence of publication bias among included studies (*P* = 0.62, Begg’s test and *P* = 0.16, Egger’s test).

## Discussion

The present meta-analysis, including 22 studies and a total of 2428 people, showed that there was no statistically significant difference in serum/plasma and hair zinc levels between patients with ADHD and their controls. There was substantial heterogeneity among included studies. However, sensitivity analysis in studies examining the circulating zinc levels showed that excluding one study^28^ changed the overall effect. Circulating levels of zinc were significantly lower in subjects with ADHD compared to healthy controls after excluding Abdelnaby's study^28^.

Zinc deficiency is involved in a variety of neurological disorders including autism, seizures, depression, and anxiety disorders^44^. However, the exact mechanism of zinc in ADHD is still unclear. Dopamine is a neurotransmitter plays a crucial role in the pathophysiology of ADHD^9^. Previous studies reported that zinc is involved in the production of melatonin which could regulate dopamine levels and homeostasis^45,46^. Dysfunction in the dopamine transporter is another pathway that contributed to the etiology of ADHD^9^. Zinc binding to the dopamine receptors inhibits the dopamine re-uptake and increases the carrier-mediated dopamine efflux^9,46^. Also, zinc is an important cofactor for several enzymes in the brain involved in the neurotransmitters and prostaglandins production^9^.

Several studies have suggested the role of inflammation and oxidative stress in the pathogenesis of ADHD^47,48^. Although subjects with ADHD have normal levels of antioxidant capacity, their reaction to oxidative stress is impaired^47^. Elevated levels of pro-inflammatory cytokines could decrease the levels of zinc in patients with ADHD through the sequestration of zinc in the liver and spleen^49^. Zinc could exert anti-oxidative and anti-inflammatory properties through the protection of sulfhydryl groups of proteins from oxidation^50^. Zinc takes part in antioxidant enzyme production and acts as a cofactor of several enzymes^50^. Also, zinc modulates the chronic inflammatory status by reducing pro-inflammatory cytokines^51^. On the other hand, zinc supplementation showed beneficial effects in the alleviation of hyperactivity symptoms in zinc-deficient ADHD subjects^52^. Moreover, 150 mg/day zinc supplementation for 12 weeks led to a significant reduction in symptoms of hyperactivity, impulsivity, and impaired socialization in patients with ADHD^53^. Although, 30 mg/day zinc supplementation showed no significant effects on primary outcomes compared to the placebo, which might be due the low dosage of zinc^54^.

Lower levels of zinc in subjects with ADHD may be attributed to the dietary zinc intake or zinc absorption^49^. Also, zinc-wasting in the urine is another possible cause of low levels of zinc in children with ADHD^55^. It has been suggested that hyperactive children have increased levels of urinary zinc and reduce levels of plasma^49^.

Sensitivity analysis showed that the exclusion of Abdelnaby's study^28^ from the analysis changed the overall effect size. The pooled analysis without mentioned study showed significant lower levels of serum/plasma zinc in subjects with ADHD compared to their controls. Indeed, the mentioned study showed a significant higher levels of serum/plasma zinc in subjects with ADHD compared to the controls. This contradictory finding could be related to several factors e.g. different study design, small sample size, the different method in zinc measurement, and high risk of bias (NOS = 6).

The present study has some limitations that should be acknowledged. We observed a significant heterogeneity among included studies that could affect the generalizability of results. However, our attempts to detect the potential source of heterogeneity through different subgroup analysis were unsuccessful. The observed heterogeneity in the present meta-analysis could be related to several factors including demographic and clinical differences, BMI, study design, adjusted models for statistical analysis, risk of bias, and methods for assessing zinc levels. Small sample sizes of individual studies are another limitation of the present study. Almost all of the included studies except one^26^ were performed on less than 200 participants. Moreover, included studies did not evaluate the dietary intake of zinc in study participants which could affect the results because that amount of zinc intake is related to the serum zinc concentration^56^. Also, many factors could affect hair zinc levels^57^, which should be taken into the interpretation of results.

## Conclusion

Present meta-analysis raises the possibility that subjects with ADHD are prone to have declined levels of zinc levels. Based on current findings screening the zinc levels at the beginning of the diagnosis in subjects with ADHD could be reasonable. Further well-designed studies are needed to clarify the role of zinc in the etiology of ADHD.
